# Unveiling the hidden arsenal: new insights into *Proteus mirabilis* virulence in UTIs

**DOI:** 10.3389/fcimb.2024.1465460

**Published:** 2024-11-13

**Authors:** Aoyu Yang, Yuchong Tian, Xiancheng Li

**Affiliations:** ^1^ Department of Urology, The Second Affiliated Hospital of Dalian Medical University, Dalian, China; ^2^ Department of Endocrinology, Shengjing Hospital of China Medical University, Shenyang, China

**Keywords:** urinary tract infections, catheter-associated urinary tract infections, virulence factors, biofilm formation, urinary stone formation, *Proteus mirabilis*

## Abstract

*Proteus mirabilis* is a Gram-negative bacterium commonly found in urinary tract infections (UTIs) and catheter-associated urinary tract infections (CAUTIs). The pathogenic mechanisms of *Proteus mirabilis* are complex and diverse, involving various virulence factors, including fimbriae, flagella, urease, polyphosphate kinase, lipopolysaccharides, cyclic AMP receptor protein, Sigma factor RpoE, and RNA chaperone protein Hfq. These factors play crucial roles in bacterial colonization, invasion, evasion of host immune responses, biofilm formation, and urinary stone formation. This paper is the first to comprehensively describe the hydrogenase system, autotransporter proteins, molybdate-binding protein ModA, and two-component systems as virulence factors in *Proteus mirabilis*, providing new insights into its pathogenic mechanisms in urinary tract infections. This review explores the mechanisms of biofilm formation by *Proteus mirabilis* and the various virulence factors involved in UTIs, revealing many newly discovered virulence factors from recent studies. These findings may offer new targets for clinical treatment of UTIs and vaccine development, highlighting the importance of understanding these virulence factors.

## Introduction

*Proteus mirabilis* is widely distributed in natural environments such as water, soil, and the gastrointestinal tracts of humans and animals (Drzewiecka, 2016; Wasfi et al., 2020). In the human gut microbiota, Proteus species constitute less than 0.05% in healthy individuals (Yatsunenko et al., 2012). Among all species of the genus Proteus, *Proteus mirabilis* is the most common cause of human infections (Jacobsen and Shirtliff, 2011). *Proteus mirabilis* can also cause osteomyelitis, mastoiditis, rheumatoid arthritis (Salehi et al., 2023; Juárez-Chairez et al., 2024), wound infections, otitis media (Leichtle et al., 2024), keratitis (Mo et al., 2021), Crohn’s disease (Zhang et al., 2021),neonatal meningitis, hemorrhagic meningoencephalitis (Senior and Sweeney, 1984; Chang et al., 2002; Phan and Lehman, 2012; Ebringer and Rashid, 2014), and hepatic encephalopathy in patients with acute liver failure (Samtoy and DeBeukelaer, 1980). Previous studies have reported that the most common type of kidney stone caused by urinary tract infections is struvite, which contains magnesium ammonium phosphate, and the most common microorganisms found in these stones are urease-producing bacteria, particularly *Proteus mirabilis* (Griffith, 1982; McLean et al., 1985; Miano et al., 2007).Furthermore, *Proteus mirabilis* has recently been implicated in the pathogenesis of Parkinson’s disease (Li et al., 2017; Choi et al., 2018). *Proteus mirabilis* is also a common cause of urinary tract infections in patients with certain anatomical or functional abnormalities of the urinary system (Odoki et al., 2019). This is particularly problematic for patients with long-term indwelling catheters (Gambrill et al., 2024; Khorasani et al., 2024), as it adheres to the catheter surface and obstructs urine flow, eventually forming a crystalline biofilm (Pelling et al., 2019). As a result, these patients are at significantly higher risk of catheter-associated urinary tract infections.

**Figure 1:**
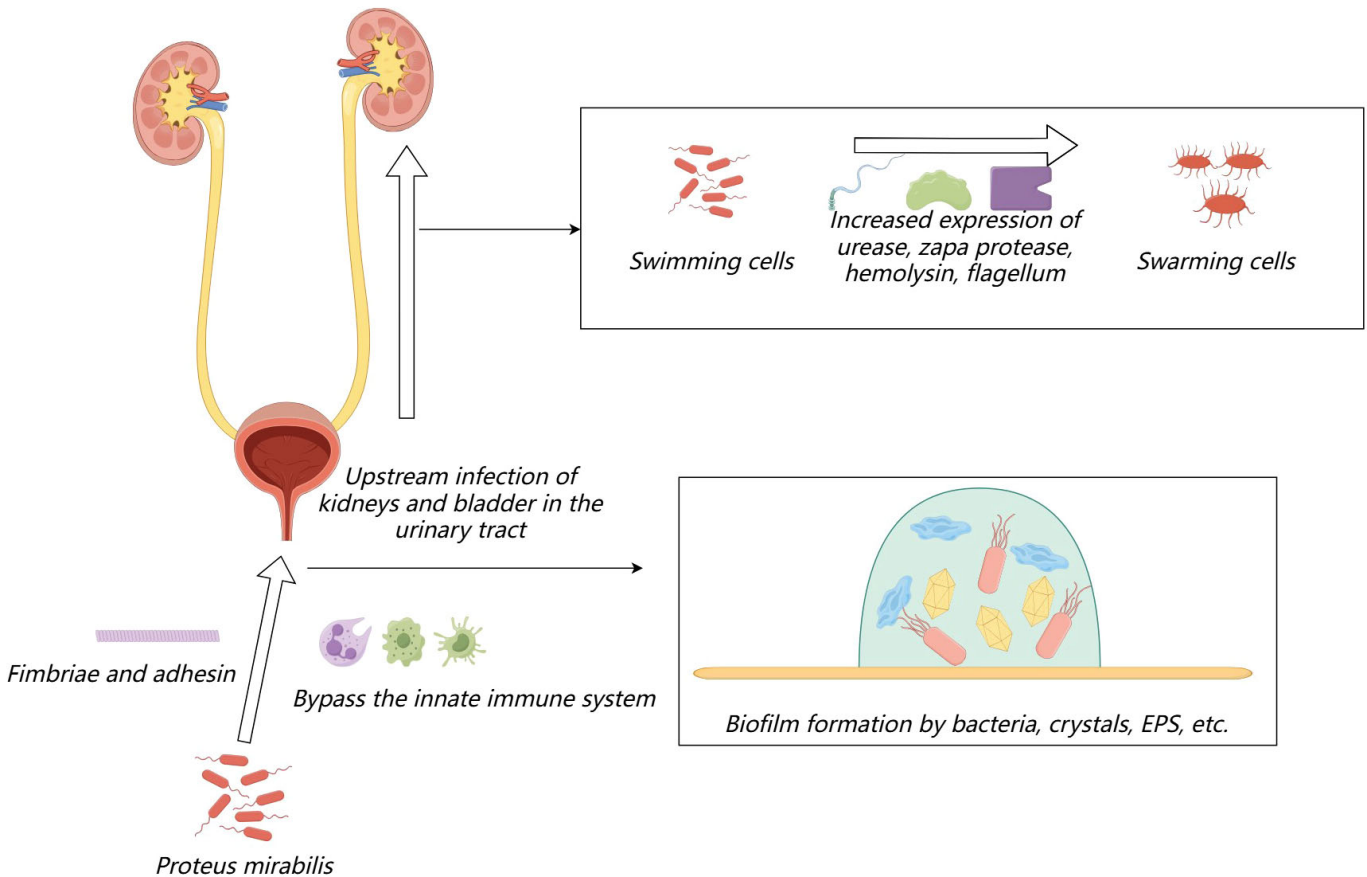
The process of urinary tract infection induced by Proteus mirabilis.

During urinary tract infections, *Proteus mirabilis* initially bypasses the host’s natural defense systems to infect the urethra, where it subsequently colonizes. It can then migrate to the bladder and further to the kidneys. The adherence to the uroepithelium or catheters relies on fimbriae and adhesins. During migration, *Proteus mirabilis* differentiates from individual short rod-shaped “swimming cells” into multicellular elongated “swarming cells.” This differentiation involves rearrangements of bacterial membrane components (Stolarek et al., 2023), increased expression of urease, ZapA protease, and hemolysin, and an increase in flagella and nucleoid-like structures that form cellular communities (Jones et al., 2004; Rather, 2005; Jacobsen et al., 2008), which plays a crucial role in kidney colonization (Allison et al., 1994). After the adherence process, *Proteus mirabilis* can form a crystalline biofilm on the surface of the host or catheter: (1) initial colonization by bacteria surrounded by a large amount of glycosylated carbohydrates, (2) formation of sheet-like microcrystalline material, (3) accumulation of dispersed crystalline substances, and (4) formation of a biofilm containing defined crystals and highly motile *Proteus mirabilis* cells (Wilks et al., 2015). The biofilm is a complex structure where bacterial cells are surrounded by extracellular polymeric substances, primarily composed of polysaccharides, proteins, extracellular DNA, glycoproteins, lipids, teichoic acids, and lipopolysaccharides (Gmiter and Kaca, 2022). The process of urinary tract infection induced by *Proteus mirabilis* is shown in **Figure 1**. Concurrently, several recently identified virulence factors also promote the progression of urinary tract infections, which I will introduce in detail in the following sections.

## Urease

*Proteus mirabilis* secretes urease, a nickel-containing enzyme located in either the cytoplasm or outer membrane of this bacterium. Urease plays a crucial role in the formation of stones and the onset of acute pyelonephritis (Mobley, 2019). Urease hydrolyzes urea into CO2 and ammonium ions, promoting crystal formation as an initiator (Schaffer and Pearson, 2015; Combaz-Söhnchen and Kuhn, 2017; Manoharan et al., 2023). The hydrolysis process produces high concentrations of ammonia, which raises the pH of the urine. The increased concentrations of ammonium, bicarbonate, and phosphate ions lead to ion precipitation within infection stones (Chakkour et al., 2024; Prywer et al., 2024; Szczerbiec et al., 2024). These stones quickly grow larger, potentially resulting in kidney disease.

The urease gene cluster in *Proteus mirabilis* consists of *ureABC* and *ureDEFG*. *ureABC* encodes the structural subunits of the apoenzyme, while *ureDEFG* encodes proteins responsible for facilitating the insertion of essential nickel ions into the catalytic site (Poore and Mobley, 2003). For urease maturation, nickel ions must be transported into the cytoplasm and incorporated into the apoenzyme. UreR is the regulatory factor responsible for activating urease operon expression, and without the *ureR* gene, urease cannot be produced (Parra and Collins, 2012). UreR is a positive regulator of urease and nickel transport, and in the presence of urea, it activates the expression of the *ure* gene cluster (Fitzgerald et al., 2024).

Studies using Alizarin Red staining and immunofluorescence to detect calcium and magnesium salts and extracellular clusters compared urease-negative *ureC* mutants with control groups. As expected, no Alizarin Red staining or extracellular clusters were observed in bladder sections from mice infected with the *ureC* mutant *Proteus mirabilis*, whereas strong Alizarin Red staining and extracellular clusters were observed in the control group. These findings underscore the critical role of urease in the formation of extracellular clusters and stone deposition by *Proteus mirabilis in vivo*. At 24 hours post-infection, the CFU of the *ureC* mutant was 23 times lower than that of the control group, although the mutant did not exhibit growth defects under various *in vitro* conditions (Schaffer et al., 2016).

Urease secreted by *Proteus mirabilis* also exhibits some non-enzymatic properties, such as promoting platelet aggregation, increasing intracellular calcium levels in mouse neuroblastoma cells, and inducing a pro-inflammatory phenotype in HEK293 cells (derived from human embryonic kidney). In the presence of urease, this results in the production of reactive oxygen species and the secretion of IL-1β and TNF-α. In addition to the toxicity caused by ammonia to urinary tract tissues, urease may also exacerbate tissue damage through other non-enzymatic mechanisms (Grahl et al., 2021). The virulence factors of *Proteus mirabilis*, along with their associated genes, related functions, and references, are summarized in **Table 1**.

**Table 1:** Virulence factors, associated genes, related functions, and references of *Proteus mirabilis*.

Virulence Factor	Associated Gene	Gene Function	References
Urease	*ureABCDEFG, ureR*	Catalyzes the breakdown of urea into ammonia and carbon dioxide, raising the pH and promoting the formation of kidney stones.	(Poore and Mobley, 2003; Parra and Collins, 2012; Schaffer and Pearson, 2015; Schaffer et al., 2016; Mobley, 2019; Grahl et al., 2021; Manoharan et al., 2023; Chakkour et al., 2024; Fitzgerald et al., 2024; Prywer et al., 2024; Szczerbiec et al., 2024)
Hydrogenase system	*hybABCDE, hyfABCDEFGHIJ*	Oxidizes hydrogen and generates a proton motive force, regulating urease activity and contributing to the formation of urinary stones, as well as bacterial colonization in the bladder and kidneys.	(Pearson et al., 2011; Learman et al., 2019; Lin and Liaw, 2020; Brauer et al., 2023)
Lipopolysaccharide	*arnA, waaF, waaC,wamD, wamA, wabN, wabH, wabG, waaQ, waaA, waaE*	Binds ions involved in stone formation, facilitates the embedding of crystals in biofilms, and regulates bacterial adaptation in the urethra and spleen.	(Breazeale et al., 2005; Aquilini et al., 2010; Prywer and Torzewska, 2019; Biondo, 2023; Clarke et al., 2023; Herout et al., 2023)
Mannose-Resistant/Proteus-like (MR/P) fimbriae	*mrpABCDEFGHJ*	Mediates bacterial adhesion and colonization on host cells, contributes to biofilm formation and urinary stone development, and regulates multiple virulence-associated genes in bacteria.	(Hay et al., 1999; Li et al., 1999; Alamuri and Mobley, 2008; Pearson et al., 2011; Bode et al., 2015; Schaffer et al., 2016; Norsworthy and Pearson, 2017; Debnath et al., 2018; Jiang et al., 2020)
ZapA Protease	*zapABCD*	Degrades host antimicrobial peptides, aiding bacterial immune evasion.	(Belas et al., 2004)
Type VI secretion system (T6SS)	*idsABCDEF, idrABCDE, tssA-Q*	Involved in self-recognition during swarming processes, contributing to bacterial adaptation during pyelonephritis and potential systemic dissemination.	(Wenren et al., 2013; Debnath et al., 2018)
Ambient temperature fimbriae (ATF)	*atfABCDEJ*	Associated with bacterial motility and adhesion, though the exact pathogenic mechanism remains unclear.	(Zunino et al., 2000; Bode et al., 2016)
Uroepithelial cell adhesin (UCA) fimbria	*ucaA*	Related to bacterial adhesion and localization in the kidneys.	(Wray et al., 1986; Tolson et al., 1997; Pellegrino et al., 2013)
Proteus mirabilis fimbriae (PMF)	*pmfACDEF*	Facilitates bladder colonization during urinary tract infections and kidney colonization during bloodstream infections.	(Zunino et al., 2003; Zunino et al., 2007)
Flagella	*flhDC, fliA, flgM*, and other genes associated with class 2 and class 3 promoters.	Involved in swimming and swarming motility, promotes the expression of pro-inflammatory chemokines in the bladder, and is associated with bacterial surface sensing capabilities.	(Chevance and Hughes, 2008; Morgenstein et al., 2010; Umpiérrez et al., 2013; Lee and Belas, 2015; Asadi Karam et al., 2018; Scavone et al., 2023)
Membrane-associated protein	*wosA*	Induces swarm cell differentiation when overexpressed, potentially related to the regulation of the flagellar complex signaling pathway.	(Hatt and Rather, 2008)
L-serine deaminase	*sdaA, sdaB*	Associated with biofilm integrity and flagellar motility.	(Zhang and Newman, 2008)
Molybdate-binding Protein ModA	*modA*	Involved in molybdate transport, linked to bacterial anaerobic respiration and biofilm formation, and regulates the expression of flagellar and fimbrial genes.	(Kumar and Park, 2018; Peng et al., 2020; Palusiak, 2022; Huang et al., 2023; Zhao et al., 2023)
Autotransporter proteins (ATs)	*pta, aipa, taap*	Promotes bacterial adhesion to host cells or extracellular matrix proteins, associated with bacterial invasion capability, cytotoxicity, serum resistance, and auto-aggregation.	(Stathopoulos et al., 2000; Henderson and Nataro, 2001; Alamuri and Mobley, 2008; Alamuri et al., 2010; Guérin et al., 2017)
Two-component systems (TCS)	*rcsB, rcsC, rcsD, rcsF, rppA, rppB, glmY, qseE, qseG, qseF, cpxR*	Involved in osmoregulation, membrane stress response, and cell differentiation, regulates flagellin synthesis, hemolysin activity, and sensitivity to polymyxin B; associated with swarming motility, flagellar promoters, biofilm formation, protease activity, and bacterial adhesion capacity.	(Brill et al., 1988; Stock et al., 2000; Takeda et al., 2001; Castanié-Cornet et al., 2006; Wang et al., 2008; Jiang et al., 2010; Howery et al., 2016; Chen et al., 2020; Lin et al., 2022; Huang et al., 2024)
Sigma factor RpoE	*rpoE, rseABC*	Maintains bacterial envelope integrity, associated with colonization ability, inhibits swarming motility, swarm cell differentiation, and swarm-associated hemolysin activity.	(De Las Peñas et al., 1997; Kanehara et al., 2002; Crouch et al., 2005; Liu et al., 2015)
Polyphosphate kinase (PPK)	*ppk*	Regulates polyphosphate synthesis, contributes to bacterial resistance to oxidative, osmotic, and thermal stress, and is associated with bacterial adhesion and invasion capabilities.	(Tzeng and Kornberg, 1998; Santoro, 2000; Nachin et al., 2005; Peng et al., 2016)
Cyclic AMP receptor protein (CRP)	*crp*	Regulates bacterial metabolism and coordinates virulence factor expression, associated with kidney colonization and stress tolerance, and is crucial for swimming and swarming motility.	(Gosset et al., 2004; Zheng et al., 2004; Shimada et al., 2011; Tsai et al., 2017)
RNA chaperone protein Hfq	*hfq*	Involved in small RNA regulation, helps resist damage caused by ROS, high temperatures, and high osmotic pressure; associated with bladder and kidney colonization, swimming and swarming motility, flagellin synthesis, hemolysin activity, and biofilm formation.	(Brennan and Link, 2007; Chao and Vogel, 2010; Richards and Vanderpool, 2011; Wang et al., 2014)

## Hydrogenase system

**Figure 2:**
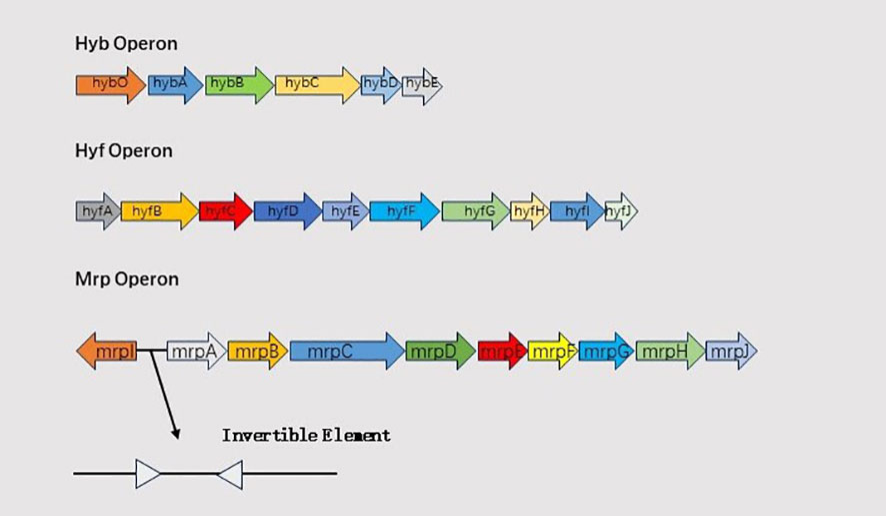
Schematic representation of hyb,hyf and mrp operon.

Formate hydrogenlyase (FHL), composed of formate dehydrogenase H (FDH) and a [NiFe] hydrogenase complex, links formate oxidation to proton reduction, facilitating H_2_ production. FhlA, a RpoN-dependent enhancer-binding protein, serves as the transcriptional activator of FHL (Schlensog et al., 1994; Self et al., 2004). The hydrogenase in FHL is a [NiFe] hydrogenase, playing a role in converting protons to hydrogen gas (Schoelmerich and Müller, 2020). In *Proteus mirabilis* HI4320, two hydrogenases are encoded: the FHL hydrogenase 2 operon (*hyb*) and hydrogenase 4 operon (*hyf*). *hyfG* encodes the [NiFe] hydrogenase large subunit required for hydrogenase activity (Noguchi et al., 2010). The primary role of *hyb* and *hyf* is to convert protons into hydrogen gas, deacidifying the environment (Schoelmerich and Müller, 2020). The *hyfH* gene is significantly upregulated in a mouse model of ascending urinary tract infection (Pearson et al., 2011), and *hyfE* has been identified as a bladder colonization adaptation factor through transposon sequencing. Schematic representation of hyb,hyf and mrp operon is shown in **Figure 2**.

In *Proteus mirabilis*, *FhlA* regulates the promoter activities of *fdhF* and *hyf*, significantly induced by formate and anaerobic conditions (Lin and Liaw, 2020). In the *hyfC* mutant, *hyfA* expression is notably higher than in the wild-type or *hyfE* mutants, suggesting that the *hyb* operon may influence the *hyf* operon, although specific mechanisms require further investigation (Brauer et al., 2023).

Deletion of *hyfG* or *fhlA* leads to reduced urease activity and urinary stone formation, with significantly lower urease activity observed in these mutants across a pH range of 5 to 8 (Lin and Liaw, 2020). However, *hybC* and *hyfE* do not affect urease activity (Brauer et al., 2023).

The structure of *hyf* resembles respiratory complex I, suggesting involvement in proton gradient formation and energy production (Schoelmerich and Müller, 2020). The rotation of flagella requires the proton motive force (PMF) (Gabel and Berg, 2003). While deletion of *hybC*, *fhlA* or *hyfG* does not impact proton motive force (PMF) phenotypes, the *hyf* system contributes to PMF under anaerobic conditions (Lin and Liaw, 2020; Brauer et al., 2023).

*FhlA* and *hyfG* are essential for *Proteus mirabilis* colonization. Mutants lacking *fhlA* or *hyfG* show significantly reduced colonization in both the bladder and kidneys compared to the wild-type strain (Lin and Liaw, 2020). Deletion of both *hybC* and *hyfE* leads to severe colonization defects in the bladder, kidneys, and spleen, while the loss of either gene alone does not affect colonization or adaptation. Studies have shown that co-culturing *Proteus mirabilis* with *Enterococcus faecalis* can increase cytotoxicity, and mice with co-infections exhibit more severe disease symptoms compared to single infections. 96% of the co-infected mice developed bacteremia (an 18% increase compared to *Proteus mirabilis* single infections), and 56% developed urolithiasis (a 30% increase compared to *Proteus mirabilis* single infections). Additionally, co-infection leads to severe bladder tissue damage and inflammation (Learman et al., 2019). When *Proteus mirabilis* is co-cultured with *Enterococcus* faecalis, the loss of both *hybC* and *hyfE* operons does not cause adaptation defects. In competitive co-culture, *hybC* and *hyfE* offer a slight adaptation advantage during the lag and early log phases, but their high expression inhibits growth in the stationary phase. Direct competition showed that the CFU of the *hyfE* mutant was higher than the *hybC* mutant, indicating that increased *hyf* expression in the *hybC* mutant leads to adaptation defects (Brauer et al., 2023).

## Lipopolysaccharide

Lipopolysaccharide, present in the outer membrane of Gram-negative bacteria, safeguards bacterial integrity and acts as a critical virulence factor. The LPS of *Proteus mirabilis* is composed of three parts: lipid A, the core region, and the O-antigen polysaccharide (Palusiak, 2013). The O-antigen exhibits the highest biological activity. Its absence can lead to various changes in the bacteria, such as increased sensitivity to antimicrobial agents, altered hydrophobicity, impaired swarming motility, adhesion to atherosclerotic plaques, and heightened sensitivity to human serum (Xue et al., 2020; Wang et al., 2021; Liu et al., 2022; Clarke et al., 2023). In struvite stones resulting from urinary tract infections involving *Proteus mirabilis*, LPS can participate in stone formation, mainly by binding ions (Prywer and Torzewska, 2019). The negatively charged polysaccharides can bind free cations in urine (such as calcium and magnesium ions), causing these ions to aggregate around the bacteria, which accelerates stone formation and incorporates them into the stone.

The *waa* gene cluster in the core region of *Proteus mirabilis* LPS includes *waaC*, which is involved in core synthesis (Aquilini et al., 2010). Inactivation of *waaC* leads to partial loss of the core region and O-antigen, resulting in reduced motility, impaired swarming, increased serum sensitivity, and significantly heightened sensitivity to antimicrobial agents (with MIC reductions up to 64-fold). Crucially, in an *in vitro* bladder model, the ability of *Proteus mirabilis* to form crystalline biofilms is significantly reduced, thereby delaying catheter blockage. This may be due to the incomplete LPS structure from *waaC* gene deletion, inhibiting crystal embedding into biofilms. Further research is warranted to explore *waaC* as a potential target for inhibiting stone formation (Clarke et al., 2023).

The modification of lipid A in LPS requires the bifunctional polymyxin resistance protein *ArnA*, allowing bacteria to resist host antimicrobial peptides (Breazeale et al., 2005). *ArnA* mutants show reduced adaptability in the urethra and spleen, with significantly lower CFU counts adhering to bladder cells (Herout et al., 2023). However, the specific mechanisms and regulatory networks of *ArnA* in *Proteus mirabilis* regarding resistance to host immunity and adaptation to environmental stress still require further exploration.

Poorly crystalline and amorphous precipitate (PCaAP) is a component of infectious urinary stones, characterized by its lack of crystallinity and amorphous nature. It includes amorphous calcium carbonate (ACC), amorphous calcium phosphate (ACP), and/or amorphous carbonated calcium phosphate (ACCP). PCaAP can aggregate with *Proteus mirabilis* and struvite, accelerating stone formation and resulting in larger stones. LPS is the primary virulence factor in *Proteus mirabilis*, promoting PCaAP aggregation. The effect of LPS on PCaAP aggregation depends on its concentration. LPS at 12 μg/ml and 50 μg/ml enhances PCaAP aggregation, while 360 μg/ml and 440 μg/ml inhibit it (Prywer and Torzewska, 2019), This inhibitory effect may be due to high concentrations of LPS coating the PCaAP particles, preventing further aggregation. However, this does not imply that increasing LPS levels would generally reduce the formation of poorly crystalline and amorphous precipitates, but rather that it influenced the aggregation process under specific experimental conditions. LPS provides favorable sites for PCaAP nucleation, and they can bind through van der Waals forces or hydrogen bonds. This interaction may lower the energy threshold for PCaAP nucleation, thereby accelerating nucleation (Yang et al., 2003). Future research focusing more on biofilm formation and bacterial surface components related to adhesion and invasion capabilities would greatly enhance our understanding and treatment of Proteus mirabilis-induced urinary tract infections (Biondo, 2023).

## Fimbriae

Fimbriae enable bacteria to adhere to various abiotic and biotic surfaces. This adhesion capability allows many fimbriae to function as virulence factors, interacting with hosts and materials (Blomfield and van der Woude, 2007). *Proteus mirabilis* contains 17 potential chaperone-usher fimbrial operons, the highest number recorded in any sequenced bacterial genome to date (Pearson et al., 2008). Four fimbrial operons have been linked to the virulence of *Proteus mirabilis*: Mannose-Resistant/Proteus-like fimbriae (MR/P), urothelial cell adhesin (UCA, also known as non-agglutinating fimbriae or NAF), *Proteus mirabilis* fimbria (PMF), and Fimbria 14. The virulence mechanisms of ambient-temperature fimbria (ATF) remain unclear. The following sections will introduce each type of fimbriae.

## Mannose-resistant/Proteus-like fimbriae

The formation of extracellular clusters by *Proteus mirabilis* is an essential early stage in urinary stone development, requiring MR/P fimbriae and urease. Thus, MR/P fimbriae are crucial for urinary stone formation induced by *Proteus mirabilis* (Schaffer et al., 2016). The *mrp* operon comprises *mrpABCDEFGHJ* (Pearson et al., 2011), with *mrpA* encoding the major structural subunit, *mrpC* encoding the outer membrane usher, *mrpD* encoding the periplasmic chaperone, *mrpB* and *mrpE-G* encoding four minor subunits, *mrpH* encoding the tip-located adhesin, and *mrpJ* encoding the transcriptional regulator (Li et al., 2002). The gene *mrpI*, separate from the *mrp* operon and independent of the *mrp* promoter, encodes a recombinase that can switch the Invertible Element (IE) from “ON” to “OFF” or from “OFF” to “ON” to control MR/P fimbriae expression (Li et al., 2002).

MrpH, located at the tip of mannose-resistant Proteus-like (MR/P) fimbriae, is essential for the assembly of MR/P fimbriae and necessary for their adhesion to surfaces (Li et al., 1999). The adhesive protein domain of MrpH features a unique folding structure with a Zn2+ binding site composed of three conserved histidine residues. MrpH relies on the binding of zinc ions for biofilm formation, and disrupting the zinc binding site can significantly inhibit the biofilm formation of *Proteus mirabilis*. Additionally, zinc ions can reversibly regulate the biofilm formation process (Jiang et al., 2020). In the future, we hope to see research targeting MrpH to explore whether MrpH can prevent biofilm formation of *Proteus mirabilis* and to investigate the specific molecular mechanisms involved.

MrpJ is a predicted helix-turn-helix (HTH) protein essential for an ascending UTI mouse model. The study found that the MrpJ mutant showed significantly reduced ability to colonize the urinary tract in mice (Norsworthy and Pearson, 2017). MrpJ can regulate multiple virulence factor genes in *Proteus mirabilis*, which are closely related to urinary tract infections and the induction of urinary stones caused by *Proteus mirabilis*. MrpJ can inhibit the flagellar gene *flhDC*, fimbriae genes *pmfA*, fim8A, and fim14A, as well as lipopolysaccharide (LPS) modification genes *pagP*, *lpxK*, and *msbB* (which are suspected to be involved in bacterial immune evasion). Additionally, it inhibits the swarming regulators *umoA* and *umoB*, the cell differentiation gene *lrhA* involved in swarming, and the gene ccm that determines cell shape with the strongest inhibitory effect (Hay et al., 1999; Bode et al., 2015). MrpJ can also promote the expression of the Pta toxin and inhibit the expression of the ZapA protease, where Pta is an autotransporter protein that promotes bacterial aggregation and can interact with bladder and renal epithelial cells (Alamuri and Mobley, 2008). The ZapA protease helps bacteria evade the immune system by cleaving antimicrobial peptides in urine (Belas et al., 2004). In addition, MrpJ can promote the expression of the T6SS system (Bode et al., 2015), which is related to self-recognition during the swarming process and contributes to bacterial adaptation during pyelonephritis and potential systemic dissemination (Wenren et al., 2013; Debnath et al., 2018). Recently, genes encoding the T6SS, such as tssA, idsA, hcp, and hcp3, have been identified as direct targets of MrpJ (Debnath et al., 2018).

*Proteus mirabilis* employs fimbriae-associated transcriptional regulators to balance these opposing processes by upregulating fimbrial adhesion while downregulating flagellar expression.

## Other fimbriae

Research on ambient temperature fimbriae (ATF) is limited, with existing studies indicating that it is not essential for infection (Zunino et al., 2000). Recent research has revealed the role of a site within the *atf* promoter, *atfJ*. *atfJ* is essential for the expression of the *atf* operon and has a dual regulatory role in motility and adhesion. However, the specific relationship between *atfJ* and the pathogenic mechanism of *Proteus mirabilis* remains unclear and requires further research for confirmation (Bode et al., 2016).

The uroepithelial cell adhesin (UCA) fimbria, also known as nonagglutinating fimbriae (NAF), was the first fimbria identified in *Proteus mirabilis* (Wray et al., 1986), also known as nonagglutinating fimbriae (NAF). NAF can mediate the attachment of *Proteus mirabilis* to surface receptors of different cells (Tolson et al., 1997). The absence of UCA fimbriae significantly impairs the adhesive ability of *Proteus mirabilis*, suggesting that UCA may play a role in its localization within the kidneys (Pellegrino et al., 2013).

PMF contributes to bladder colonization during urinary tract infections and kidney colonization during bloodstream infections (Zunino et al., 2003). Both PMF and MR/P fimbriae have specific and cumulative effects in urinary tract infections caused by *Proteus mirabilis* (Zunino et al., 2007).

For ATF fimbriae, different scholars have demonstrated that it does not play a virulence role in urinary tract infections caused by *Proteus mirabilis*. However, experiments conducted by Paola Scavone and colleagues confirmed that UCA mutants and PMF mutants produced more extracellular matrix after two days of biofilm assessment compared to the control group. After seven days of cultivation, they produced the highest total bacterial volume (Scavone et al., 2016). Although some studies have addressed this field, the functions of UCA fimbriae and PMF and their roles in *Proteus mirabilis* biology have not been fully explored. Thus, further research on UCA fimbriae and PMF is essential to uncover their potential mechanisms in bacterial growth, environmental adaptation, and host interactions.

## Flagella

The primary functions of bacterial flagella are motility and pathogenicity. In *Proteus mirabilis*, flagella influence both swimming and swarming motility, aiding in ascension to the kidneys and migration into the bloodstream (Asadi Karam et al., 2018; Scavone et al., 2023).

*Proteus mirabilis* mutants lacking flagellin cannot swim or swarm on LB agar plates and cannot migrate along latex and silicone catheters. Their biofilm formation ability in LB broth is significantly impaired (p < 0.0001) (Scavone et al., 2023).

In *Proteus mirabilis*, flagella expression is regulated through a three-tiered hierarchy (Chevance and Hughes, 2008). The *flhDC* operon, expressed from class 1 promoters, encodes the master flagellar transcriptional regulator complex FlhD4C2. FlhD4C2 activates class 2 promoters, which transcribe genes including *fliA* (encoding the flagellar-specific sigma factor σ28), *flgM* (encoding the anti-σ28 factor), and genes encoding proteins that comprise the HBB complex and the type III flagellar secretion system. *FliA* is essential for transcribing class 3 promoters, which include genes necessary for flagellum production, stator complexes, and chemotaxis (Morgenstein et al., 2010).

Flagellin promotes the expression of pro-inflammatory chemokines in the bladder, leading to inflammation and recruiting leukocytes to the bladder epithelium (Umpiérrez et al., 2013).

The *fliL* gene is the first gene in the class 2 operon *fliLMNOPQR*, which is controlled by FlhD4C2 and *FliA*. Research has found that bacteria lacking *fliL* can still swarm. However, the deletion of *fliL* alters *Proteus mirabilis*’s surface sensing (i.e., Δ*fliL* cells are more sensitive to low-viscosity agar), enhances the differentiation of *Proteus mirabilis* swarm cells, and makes the motility of cells lacking *fliL* temperature-dependent (Lee and Belas, 2015).

The *wosA* gene encodes a membrane-associated protein that can induce swarm cell differentiation when overexpressed. The transcription levels of the *flhDC* operon and *flaA* are upregulated with *wosA* overexpression, suggesting that *wosA* may play a role in the signaling pathway of the flagellar motility switch complex (Hatt and Rather, 2008).

The *sdaA* and *sdaB* genes encode enzymes that catalyze the deamination of L-serine (Zhang and Newman, 2008), which serves as a carbon and nitrogen source for metabolism in *Proteus mirabilis*, supporting its growth and motility (Brauer et al., 2019). When both genes are absent, biofilm integrity is compromised, proton motive force is disrupted, and the bacteria exhibit severe defects in flagellar motility (Brauer et al., 2022).

## Molybdate-binding protein ModA

Molybdenum (Mo) is a transition metal and a vital trace element for nearly all living organisms (Kumar and Park, 2018). The *modA* gene encodes the molybdate-binding protein ModA, which binds molybdate (MoO42−) with high affinity for its transport. In bacteria, molybdate is transported and incorporated into a molybdopterin precursor before the synthesis of molybdoenzymes, which play a crucial role in anaerobic respiration. ModA influences the transport of molybdate in *Proteus mirabilis*, affecting the synthesis of molybdoenzymes and, consequently, bacterial anaerobic respiration. The deletion of *modA* enhances bacterial aggregation and motility (Huang et al., 2023), which is consistent with previous research showing significant upregulation of the *modA* gene in *Proteus mirabilis* strains with migration defects (Peng et al., 2020). Additionally, the absence of ModA upregulates several genes in the flagellar assembly pathway, such as *flhB*, while downregulating multiple genes in the pilus assembly and bacterial adhesion pathways, including *mrpA*, *papA*, *fimF*, *fimC*, and *fimA*. Although bacterial aggregation and motility increase under anaerobic conditions, biofilm formation decreases. This paradox is attributed to the downregulation of the *mrpA* gene, triggered by the deletion of *modA*, which is known to contribute to *in vitro* biofilm formation via MR/P fimbriae. The downregulation of fimbrial genes, which hinders bacterial adhesion and invasiveness, is likely a key factor in the reduction of biofilm formation. We propose that biofilm formation is a complex process regulated by various factors, such as environmental conditions, nutrient availability, signaling pathways, and bacterial interactions. Additionally, *modA* is directly related to bacterial metabolism, and its deletion may disrupt specific metabolic pathways, leading to the inhibition of biofilm formation. Interestingly, transcriptomic profiling and RT-qPCR results from Δ*modA* and DP2019 strains showed significant upregulation of the *hyfB* and *fdhF* genes, which are part of the hydrogenase system mentioned earlier (Huang et al., 2023). However, no studies have yet clarified the potential link between *modA* and the regulation of hydrogenase genes. Therefore, we hope that future research will focus on exploring this connection and determining whether other regulatory factors or signaling pathways are involved in mediating this process. We believe that such studies will offer a more comprehensive understanding of ModA’s role in bacterial metabolism.

Understanding the mechanisms of ModA is crucial for developing new therapeutic strategies against harmful pathogens. Current research on ModA is still limited, and many of its mechanisms remain unclear. Studies on *Enterococcus* ModA (KpModA) have explored high-resolution anaerobic anion and molybdate-binding crystal structures, detailing interactions between ModA and molybdate and tungstate anions. This foundational research provides a pathway for targeting ModA to inhibit *Klebsiella pneumoniae* virulence (Zhao et al., 2023). Given that both *Klebsiella pneumoniae* and *Proteus mirabilis* are uropathogens and share many similarities (Palusiak, 2022), further investigation into ModA in *Proteus mirabilis* could yield valuable insights. However, such studies have not yet been conducted. Expanding this research could significantly advance our understanding and control of these pathogens.

## Autotransporter proteins

Autotransporter proteins (ATs) play critical roles in urinary tract infections by promoting bacterial adhesion to host cells or extracellular matrix proteins, host cell invasion, cytotoxicity, serum resistance, and autoaggregation (Stathopoulos et al., 2000; Henderson and Nataro, 2001). Trimeric autotransporter (AT-2) proteins form an AT subfamily with similar structural features, and their passenger domains primarily consist of adhesins, proteases, or esterases (Guérin et al., 2017).The autotransporter protein Pta, identified and named in 2008, has its cytotoxicity associated with its α-domain (Pta-α), which induces actin depolymerization and disrupts nuclear membrane stability. The cytotoxicity and autoaggregation ability of Pta mutants are significantly reduced (Alamuri and Mobley, 2008). Praveen Alamuri et al. first demonstrated two AT-2 proteins in *Proteus mirabilis*: *AipA* and *TaaP*. Like other AT-2 proteins, they exhibit high affinity for extracellular matrix proteins. In their study, *AipA* demonstrated host cell invasion while *TaaP* showed autoaggregation (Alamuri et al., 2010). Despite these findings, research on autotransporter proteins in *Proteus mirabilis* remains limited. Future studies could focus on more virulence-related experiments on these proteins or identify additional ATs and AT-2s. Overall, this field holds significant research potential, and deepening our understanding could pave the way for innovative therapeutic strategies.

## Two-component systems

Two-component systems (TCS) play essential roles in urinary tract infections caused by *Proteus mirabilis* (Huang et al., 2024). They regulate bacterial processes like osmoregulation, membrane stress response, and cell differentiation. TCS consists of a sensor histidine kinase (HK) and a response regulator (RR) (Stock et al., 2000), with the HK detecting environmental changes and triggering signal transduction through autophosphorylation. The RR then adjusts downstream gene expression to help the bacteria adapt. Understanding TCS mechanisms in *Proteus mirabilis* could lead to targeted therapies for urinary tract infections.

The Rcs phosphorelay includes *RcsF*, *RcsC*, *RcsD*, and *RcsB* (Brill et al., 1988; Howery et al., 2016). *RcsF* senses membrane stress and activates *RcsC*, which initiates a phosphate transfer to *RcsB* through *RcsD*, activating downstream pathways (Takeda et al., 2001; Castanié-Cornet et al., 2006).

The response regulator *RppA* in *Proteus mirabilis* regulates flagellin synthesis, hemolysin activity, and sensitivity to polymyxin B (PB). Knockout of *RppA* increases sensitivity to PB, alters LPS, and reduces differentiation (Wang et al., 2008). *RppA* also controls *pmrI* expression, and *pmrI* knockout mutants show similar changes in PB sensitivity, biofilm formation, and resistance to imipenem (Jiang et al., 2010).

A regulatory factor of the TCS, QseF, participates in the regulation of swarming motility in *Proteus mirabilis* through *GlmY*, an sRNA of *Proteus mirabilis*. The *glmY-qseE-qseG-qseF* genes form an operon. Mutants of *glmY* and *qseF* exhibit reduced swarming motility and related phenotypes, including shorter cells, fewer flagella, significantly lower *flhDC* promoter activity, increased *rcsB* mRNA levels, and significantly reduced *cheA* mRNA levels (Lin et al., 2022).

CpxR, a key regulator in the TCS, controls biofilm formation via the *zapABCD* operon. Mutants of *zapD* and *cpxR* show reduced biofilm formation, protease activity, and adhesion. CpxR also responds to copper, enhancing biofilm formation through MR/P fimbriae, ZapA, eDNA, and EPS (Chen et al., 2020).

## Sigma factor RpoE

RpoE, as an alternative sigma factor, is crucial for maintaining the cell envelope integrity of Gram-negative bacteria (De Las Peñas et al., 1997). The *rpoE* gene is co-transcribed with genes encoding the anti-sigma factor *rseA* and the auxiliary proteins *rseB* and *rseC* (Kanehara et al., 2002; Crouch et al., 2005). Liu et al. first introduced the function of RpoE in *Proteus mirabilis*, revealing its ability to inhibit swarming motility, swarming cell differentiation, and swarming-associated hemolysin activity. RpoE affects mrp expression by regulating *mrpI* (the mrp operon was previously described). Mutation of *rpoE* increases the cytotoxicity of *Proteus mirabilis*, significantly impairing the bacteria’s colonization ability and resulting in more immune cell infiltration in the bladder and kidneys during the early stages of infection. Additionally, RpoE can sense urea in the host urethra and polymyxin B, both of which can induce RpoE expression (Liu et al., 2015).

## Polyphosphate kinase

Polyphosphate kinase (PPK), encoded by the *ppk* gene, is a key enzyme involved in the synthesis of inorganic polyphosphate (polyP) from ATP and is highly conserved across bacterial species (Tzeng and Kornberg, 1998). The study by Peng revealed the role of PPK in *Proteus mirabilis*, demonstrating its ability to promote bladder inflammation. PPK mutants lack resistance to oxidative, osmotic, and heat stress, exhibit reduced adhesion and invasion capabilities to urothelial cells, and show significantly decreased colonization in the bladder. Their swarming motility and biofilm formation abilities are also significantly weakened. Additionally, PPK mutants exhibit downregulated expression of universal stress protein G and heat shock proteins (Peng et al., 2016), both of which are important for protecting cells from stress (Santoro, 2000; Nachin et al., 2005).

## Cyclic AMP receptor protein

Cyclic AMP receptor protein (Crp) is a transcriptional regulator that modulates bacterial metabolism and coordinates the expression of virulence factors through a process known as carbon catabolite repression (CCR) (Gosset et al., 2004; Zheng et al., 2004; Shimada et al., 2011). The study by Yi-Lin Tsai et al. first revealed the role of Crp in *Proteus mirabilis*, showing that Crp activity is regulated by sugar content within the host and may be associated with urinary tract infections. Compared to the wild-type strain, crp mutants showed increased kidney colonization, decreased *flhDC* expression, and almost no swimming or swarming motility. The crp mutants also exhibited increased adhesion to renal epithelial cells, higher pmp fimbriae mRNA expression, enhanced survival rates within macrophages, increased stress tolerance, and elevated expression of the sigma factor RpoS (Tsai et al., 2017).

## RNA chaperone protein Hfq

**Figure 3:**
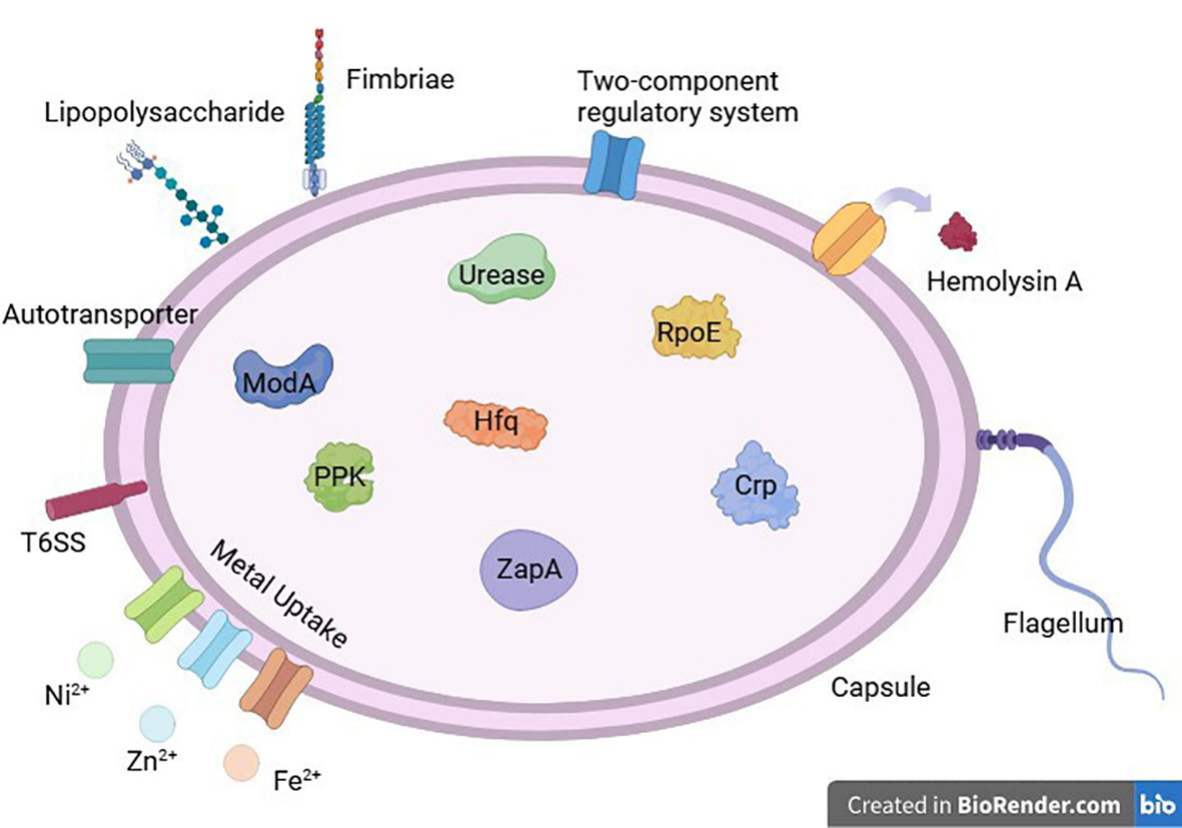
Various virulence factors of Proteus mirabilis.

Hfq, a bacterial RNA chaperone, can bind small RNAs (sRNAs) and mRNAs, participating in the riboregulation of different genes (Brennan and Link, 2007). It is also associated with stress responses, iron homeostasis, and other bacterial physiological activities (Chao and Vogel, 2010; Richards and Vanderpool, 2011) The study by Wang et al. first revealed the role of Hfq in *Proteus mirabilis*. Hfq helps resist damage caused by reactive oxygen species (ROS), high temperatures, and high osmotic pressure. Compared to the wild-type, Hfq mutants have impaired colonization ability in the bladder and kidneys, and promote the production of the key antibacterial cytokines IL-8 and MIF by urothelial cells. They also exhibit reduced swimming and swarming motility, fewer flagella, significantly decreased hemolysin activity, and significantly reduced biofilm formation. Additionally, Hfq mutants show impaired adhesion and invasion capabilities to urothelial cells and increased promoter activity of RpoE (as previously mentioned). Hfq mutants also lock the expression of the *mrp* operon, which controls MR/P fimbriae expression, in the off state (Wang et al., 2014). Various virulence factors of *Proteus mirabilis* is shown in **Figure 3**.

## Conclusion

This article provides comprehensive and detailed information on the specific mechanisms of virulence factors in *Proteus mirabilis*-induced urinary tract infections. However, there is still much to be explored regarding the changes in the expression of virulence factors during the transition from swimming to swarming motility. The interactions between different virulence factors within *Proteus mirabilis* remain unclear. The latest research indicates that *Proteus mirabilis* encodes multiple secretion system-related genes, but the specific functions of most of these systems remain largely unexplored (Tatarenkov et al., 2024). Furthermore, the host urinary tract contains multiple bacterial species, and biofilm formation involves interactions among them. Therefore, further investigation is essential to fully understand the pathogenic capacity of *Proteus mirabilis* in urinary tract infections and its interactions within the host microbiome.
